# Mucosal sensitization to German cockroach involves protease-activated receptor-2

**DOI:** 10.1186/1465-9921-11-62

**Published:** 2010-05-24

**Authors:** Kristen Page, John R Ledford, Ping Zhou, Krista Dienger, Marsha Wills-Karp

**Affiliations:** 1 Department of Pediatrics, Cincinnati Children's Hospital Medical Center, Cincinnati, OH USA; 2 Department of Pediatrics, University of Cincinnati, Cincinnati, Ohio USA

## Abstract

**Background:**

Allergic asthma is on the rise in developed countries. A common characteristic of allergens is that they contain intrinsic protease activity, and many have been shown to activate protease-activated receptor (PAR)-2 *in vitro*. The role for PAR-2 in mediating allergic airway inflammation has not been assessed using a real world allergen.

**Methods:**

Mice (wild type or PAR-2-deficient) were sensitized to German cockroach (GC) feces (frass) or protease-depleted GC frass by either mucosal exposure or intraperitoneal injection and measurements of airway inflammation (IL-5, IL-13, IL-17A, and IFNγ levels in the lung, serum IgE levels, cellular infiltration, mucin production) and airway hyperresponsiveness were performed.

**Results:**

Following systemic sensitization, GC frass increased airway hyperresponsiveness, Th2 cytokine release, serum IgE levels, cellular infiltration and mucin production in wild type mice. Interestingly, PAR-2-deficient mice had similar responses as wild type mice. Since these data were in direct contrast to our finding that mucosal sensitization with GC frass proteases regulated airway hyperresponsiveness and mucin production in BALB/c mice (Page et. al. 2007 Resp Res 8:91), we backcrossed the PAR-2-deficient mice into the BALB/c strain. Sensitization to GC frass could now occur via the more physiologically relevant method of intratracheal inhalation. PAR-2-deficient mice had significantly reduced airway hyperresponsiveness, Th2 and Th17 cytokine release, serum IgE levels, and cellular infiltration compared to wild type mice when sensitization to GC frass occurred through the mucosa. To confirm the importance of mucosal exposure, mice were systemically sensitized to GC frass or protease-depleted GC frass via intraperitoneal injection. We found that removal of proteases from GC frass had no effect on airway inflammation when administered systemically.

**Conclusions:**

We showed for the first time that allergen-derived proteases in GC frass elicit allergic airway inflammation via PAR-2, but only when allergen was administered through the mucosa. Importantly, our data suggest the importance of resident airway cells in the initiation of allergic airway disease, and could make allergen-derived proteases attractive therapeutic targets.

## Introduction

Allergic asthma is a chronic airway disorder characterized by airway inflammation, increased airway reactivity, and increased mucus production. While there is a genetic predisposition for asthma, this cannot account for the significant increase in asthma prevalence over the past 20 years. Environmental factors, including house dust mite (HDM) and cockroach (CR) exposure [1], play a significant role in allergic airway disease. One characteristic that many of these allergens, including cockroach, HDM, fungi, pollen and cat [2-7] contain proteolytic activity. The mechanism by which proteases modulate the immune system to initiate or maintain allergic airway disease is currently unclear.

We recently addressed the role of active serine proteases in GC frass on regulating allergic airway inflammation in a murine model. We found that airway hyperresponsiveness to acetylcholine and mucin production were significantly decreased when mice were exposed to protease-depleted GC frass compared to protease-containing GC frass [8]. Other studies have supported the role of proteases in modulating allergic airway disease. For example, removal of proteases from either *A. fumigatus *[9], American cockroach Per a 10 antigen [10], Epi p1 antigen from the fungus *Epicoccum purpurascens *[11]or Cur 11 antigen from the mold *Curvularia Iunata *[12] decreased airway inflammation and airway hyperresponsiveness in mouse models. These studies did not investigate mechanism(s) by which proteases mediated their effects.

Some reports have shown that protease activity in allergens may alter airway epithelial integrity by increasing epithelial permeability [13,14]. However, the proteases can also mediate biological effects through the activation of protease-activated receptors (PARs). PARs (-1, -2, -3, -4) are a family of proteolytically activated G-coupled receptors which, when activated, initiate a signal transduction pathway leading to transcriptional regulation. Of particular interest is PAR-2, which has been implicated in allergic diseases. PAR-2 is expressed by many cells in the lung, including airway epithelial cells [15], alveolar macrophages [16], fibroblasts [17], and mast cells [18]. Common allergens including HDM, cockroach and mold have been shown to activate PAR-2, resulting in increased cytokine production by airway epithelial cells [15,19,20]. Recently, activation of PAR-2 was also shown to increase the expression of thymic stromal lymphopoietin (TSLP) [21], which activates dendritic cells to polarize naïve T cells to Th2 cells. Collectively, these data suggest that proteases, through their activation of PAR-2, may link the innate and adaptive immune responses.

To date, only a few studies have investigated the importance of PAR-2 in modulating allergic airway disease. Mice sensitized and challenged with ovalbumin (OVA) along with PAR-2 activating peptides promoted allergic sensitization [22]. Sensitization by intraperitoneal injection of OVA bound to alum followed by OVA aerosol challenge showed that PAR-2-deficient mice had less cellular infiltration than wild type mice [23]. In the same study, mice which overexpressed PAR-2 when sensitized and challenged with OVA showed an increase in AHR compared to wild type mice. These studies suggest that PAR-2 may act as an adjuvant in allergic airway disease. Importantly though, these studies investigated the effect of PAR-2 activation following systemic (OVA-induced) inflammation. In this report, we investigate the role of PAR-2 activation by a real world, protease-containing allergen and we will investigate the role of proteases in the initiation of allergic responses.

## Materials and methods

### Cockroach frass

Fecal remnants (frass) were collected from German cockroaches (*Blattella germanica*) and reconstituted as previously described [24]. The frass preparation was frozen in aliquots and used throughout the entire experiment. Protease levels were measured by Azocoll assay and determined to be 19 μg/mg frass [25]. Azocoll is an insoluble protein-dye conjugate hydolysed by serine proteases [26]. A standard curve was generated by digestion of Azocoll with known concentrations of subtilisin (Type VIII protease, Sigma Chemical, St. Louis MO). We found that treatment with serine protease inhibitors aprotinin and PMSF inhibited GC frass protease activity by 80%, while cysteine protease inhibitors E64 and leupeptin had no effect [2].

### Animals

Balb/c and PAR-2-deficient mice were obtained from Jackson Laboratory (Bar Harbor, ME). The PAR-2-deficient mice were on the C57BL/6 background, which has been documented to be the least responsive to allergen exposure via the airway [8,27], thus necessitating the need to backcross these mice onto the Balb/c background. ThePAR-2 mice used in this study are on the Balb/c background. These studies conformed to the principles for laboratory animal research outlined by the Animal Welfare Act and the Department of Health, Education, and Welfare (National Institutes of Health). These studies were approved by the Cincinnati Children's Hospital Medical Center Institutional Animal Care and Use Committee.

### Sensitization and challenge protocols

**Figure 1:**
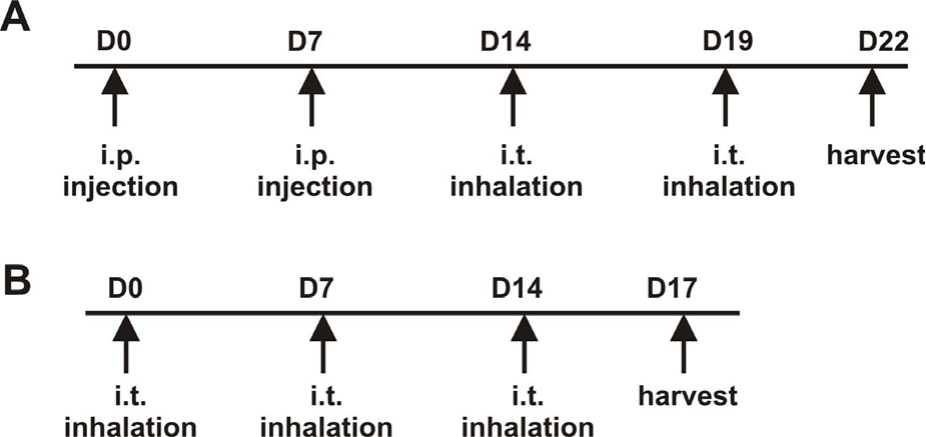
**Sensitization and challenge protocols**. A. Mucosal allergen sensitization. B. Systemic allergen sensitization.

For systemic allergen sensitization, mice were immunized with PBS or 10 μg/ml GC frass bound to alum (Imject Alum; Pierce Biotechnology, Rockford, IL) on day 0 and 7, followed by intratracheal inhalation challenges with GC frass (40 μg/40 μl) on days 14 and 19. Mice were harvested on day 22 (Figure 1A). Intratracheal inhalations are performed on anesthetized mice (45 mg/kg ketamine and 8 mg/kg xylazine) suspended on a 60 degree incline board. With the tongue gently extended, a 40 μl aliquot of PBS or GC frass is placed in the back of the oral cavity and aspirated by the mouse [27]. For airway administration of allergen, mice were given three intratracheal inhalation challenges of PBS (40 μl) or GC frass (40 μg/40 μl) on days 0, 7, and 14 and harvested on day 17 (Figure 1B).

### Airway hyperresponsiveness measurements

Allergen-induced AHR was determined as we have previously described [28]. Briefly, mice were anesthetized 72 hours after the last GC frass exposure, intubated and ventilated at a rate of 120 breaths per minute with a constant tidal volume of air (0.2 ml), and paralyzed with decamethonium bromide (25 mg/kg). After establishment of a stable airway pressure, 25 μg/kg weight of acetylcholine was injected i.v. and dynamic airway pressure (airway pressure time index [APTI] in cm-H_2_O × sec^-1^) was followed for 5 minutes.

### Serum IgE

Animals were bled and serum isolated for total IgE levels using antibodies from BD Biosciences (San Diego, CA).

### Assessment of airway inflammation

Lungs were lavaged thoroughly with 1 ml of Hanks balanced salt solution without calcium or magnesium. The lavage fluid was centrifuged (1,800 rpm for 10 min), the supernatant was removed for cytokine analysis and immediately stored at -80°C. Total cell numbers were counted on a hemocytometer. Slides of BAL cells were prepared using a Cytospin II (Shandon Thermo, Waltham, MA) and were stained with Diff-Quick (Thermo Electron Corporation, Pittsburg, PA) solution for differential cell counting.

### Cytokine production

Liberase/DNase I digests of the lung were prepared to obtain single lung cell suspensions. Single cell suspensions (2.5 × 10^5^) were incubated for 72 hours in culture medium (RPMI) or in RPMI treated with Conconavalin A (ConA; 10 μg/ml) and supernatants were analyzed by ELISA for Th2 cytokine expression as previously described [29]. IL-17A levels were determined in media without ConA.

### Histology

Whole lungs were removed and formalin fixed. Lungs were embedded in paraffin, sectioned, and stained with haematoxylin and eosin (H & E) and Periodic Acid Schiff (PAS). To quantify mucin production, we counted airways and determined the percentage of mucin stained airways (mean ± SEM; n = 3 slides per condition). Next, we picked representative airways and counted total and mucin positive cells in that airway and determined the percentage of mucin positive cells (mean ± SEM; n = 5 airways per condition).

### Statistical analysis

When applicable, statistical significance was assessed by one-way analysis of variance (ANOVA). Differences identified by ANOVA were pinpointed by Student-Newman-Keuls' multiple range test.

## Results

### PAR-2-deficient mice sensitized with GC frass by intraperitoneal injection does not alter experimentally-induced asthma in a mouse model

**Figure 2:**
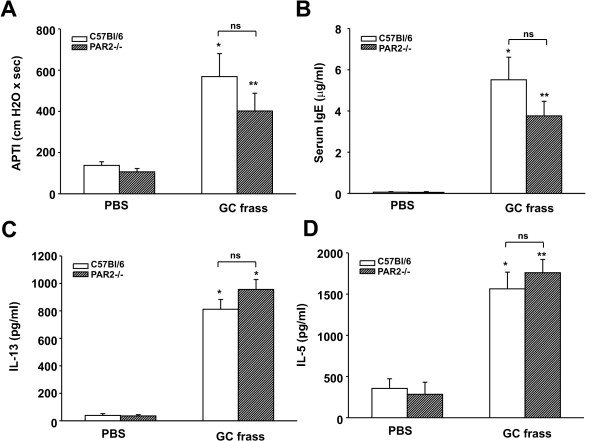
**GC frass-induced experimental allergic asthma in C57/BL6 wild type and PAR-2-deficient mice**. Wild type and PAR-2-deficient mice were sensitized on day 0 and 7 with an intraperitoneal injection of 100 ug/ml PBS or GC frass with alum (10 μg/ml). On days 14 and 19, an intratracheal inhalation was performed using PBS (40 μl) or GC frass (40 μg). On day 22, mice were anesthetized acetylcholine was injected after establishment of a stable airway pressure. In all cases the data are expressed as mean ± SEM and represent 9-10 mice per group and statistical significance determined by ANOVA. A. AHR was measured as airway pressure time index (APTI) in cm-H_2_O × sec ^-1 ^(compared to PBS *p < 0.001 and ** = 0.016). B. Serum IgE levels were analyzed by ELISA (compared to PBS *p < 0.001, **p = 0.002). C+D. Lungs from the mice were excised, and cells dissociated and maintained in a single suspension culture for 3 days in the presence of Con A (10 μg/ml). Supernatants were removed and ELISAs were run. C. IL-5 levels (compared to PBS *p < 0.001). D. IL-13 levels (compared to PBS *p < 0.001).

To determine the role of PAR-2 in mediating experimentally-induced allergic airway inflammation, we used commercially available PAR-2-deficient mice which were on a C57Bl/6 background. Since C57Bl/6 mice are the least responsive mice and do not respond well to sensitization by intratracheal inhalation [8,27], we performed the sensitization by intraperitoneal injection of GC frass bound to alum and then challenged mice via intratracheal inhalation (Figure 1A). In the wild type mice, GC frass significantly increased airway responsiveness to cholinergic agents (Figure 2A), increased serum IgE levels (Figure 2B), and increased the Th2 cytokines IL-5 and IL-13 (Figure 2C and 2D). Importantly, in the PAR-2-deficient mice, there was no significant alteration of any airway inflammatory parameters tested. GC frass induced significant recruitment of macrophages, eosinophils, neutrophils and lymphocytes into the BAL fluid (Table 1); however these levels were unaltered in PAR-2-deficient mice. Histological examination of the wild type or PAR-2-deficient mouse lungs following GC frass treatment showed dense perivascular and peribronchiolar infiltrates compared to PBS treatment (data not shown). The levels of cellular infiltrates were not different between wild type or PAR-2-deficient mice following GC frass exposure. In addition, abundant mucin was detected in the epithelial cells of wild type and PAR-2-deficient mice exposed to GC frass (data not shown). Importantly, PAR-2-deficiency had no significant effect on any of the parameters tested, suggesting that PAR-2 did not play a role in mediating allergic airway inflammation. This finding was in conflict with our previous study showing that GC frass proteases played a role in regulating airway hyperresponsiveness and mucin production [8]. Interestingly both our lab [30] and others [31] have shown that GC frass proteases cleave and activate PAR-2 in vitro; thus it is possible that the route of sensitization is an important factor in protease-PAR-2 mediated allergic airway inflammation.

**Table 1:** Differential cell count in BAL fluid of wild type and PAR-2-deficient mice.

	Mac	Epi	Eos	Neut	Lymph
**C57-PBS**	5.2 ± 1.0	5.5 ± 1.9	0.03 ± 0.02	0.02 ± 0.01	0.09 ± 0.03

**PAR2-/- PBS**	5.1 ± 1.4	7.3 ± 2.4	0.04 ± 0.02	0.05 ± 0.02	0.1 ± 0.04

**C57-frass**	35.1 ± 11	16.9 ± 7.1	11.7 ± 3.4	17 ± 7.9	23.4 ± 7.9

**PAR2-/- frass**	32.8 ± 9.9	8.6 ± 4.2	19.2 ± 5.6	14.1 ± 6.7	21.0 ± 7.0

Wild type (C57Bl/6) or PAR-2-deficient mice were given intraperitoneal injections of PBS or GC frass with alum on day 0 and 7. Intratracheal inhalations of PBS or GC frass were performed on days 14 and 19. On day 22, BAL fluid was harvested and differential cell counts performed. These data represent 9 mice per group and are expressed as mean ± SEM of cell number × 10^4^. Cell counts were statistically significant between GC frass and PBS exposed mice (*p < 0.05, as determined by ANOVA). There was no statistically significant difference between mice C57Bl/6 and PAR-2-deficient mice exposed to GC frass

### PAR-2-deficient mice sensitized with GC frass by intratracheal inhalation attenuated experimentally-induced asthma in a mouse model

**Figure 3:**
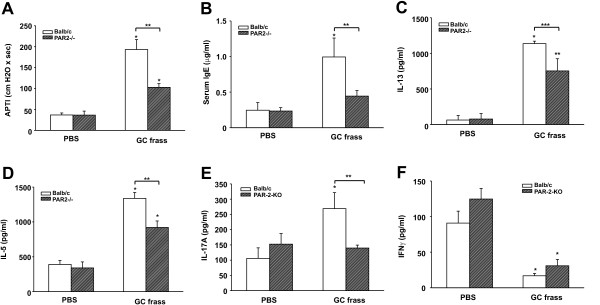
**GC frass-induced experimental allergic asthma in wild type and PAR-2-deficient mice**. Balb/c wild type and PAR-2-deficient mice were challenged by intratracheal inhalation on day 0, 7, and 14 with PBS or GC frass (40 μg). On day 17, mice were anesthetized and acetylcholine was injected after establishment of a stable airway pressure. In all cases the data are expressed as mean ± SEM and represent 4-5 mice per group and statistical significance determined by ANOVA. A. AHR was measured as airway pressure time index (APTI) in cm-H_2_O × sec ^-1 ^(compared to PBS * p < 0.001, compared to GC frass **p = 0.014). B. Serum IgE levels were analyzed by ELISA (compared to PBS *p = 0.011, compared to GC frass **p = 0.024). C-F. Lungs from the mice were excised; cells dissociated and maintained in a single suspension culture for 3 days in the presence of Con A (10 μg/ml). C. IL-13 levels (compared to PBS *p < 0.001, **p = 0.004, compared to GC frass ***p = 0.041). D. IL-5 levels (compared to PBS *p < 0.001, compared to GC frass **p = 0.004). E. IL-17A levels F. IFNγ levels (compared to PBS *p < 0.05).

**Figure 4:**
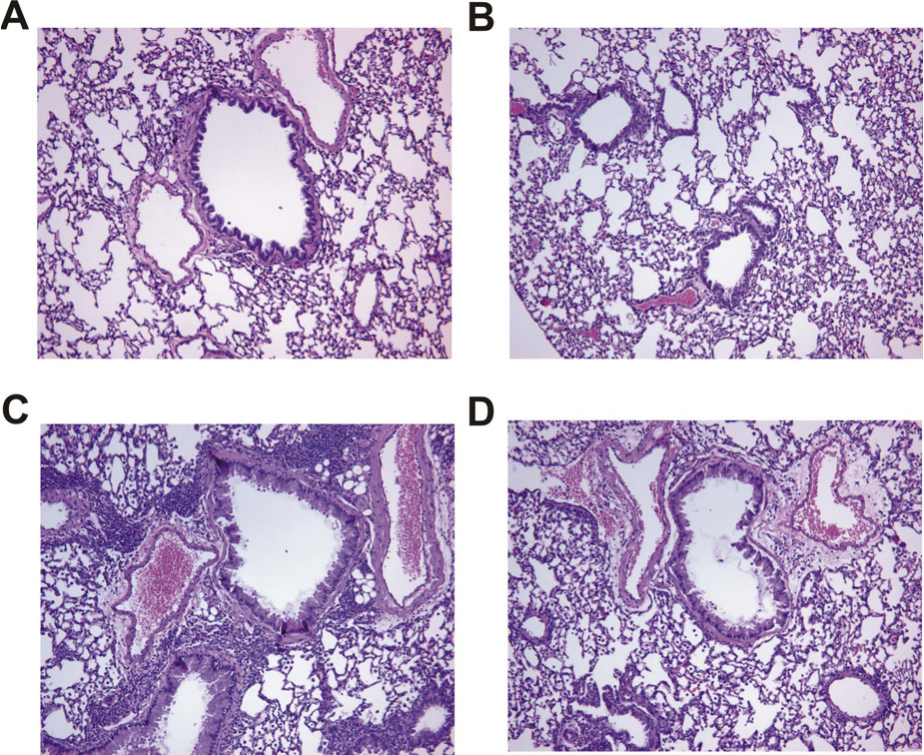
**Histological assessment of lung sections from PBS or GC frass exposed wild type and PAR-2-deficient Balb/c mice**. Haematoxylin and eosin (H&E) staining of sectioned lungs from PBS (A and B) or GC frass (C and D) treated wild type (A and C) or PAR-2-deficient (B and D) mice. Representative slides are shown of sections from 4-5 mice per group.

**Figure 5:**
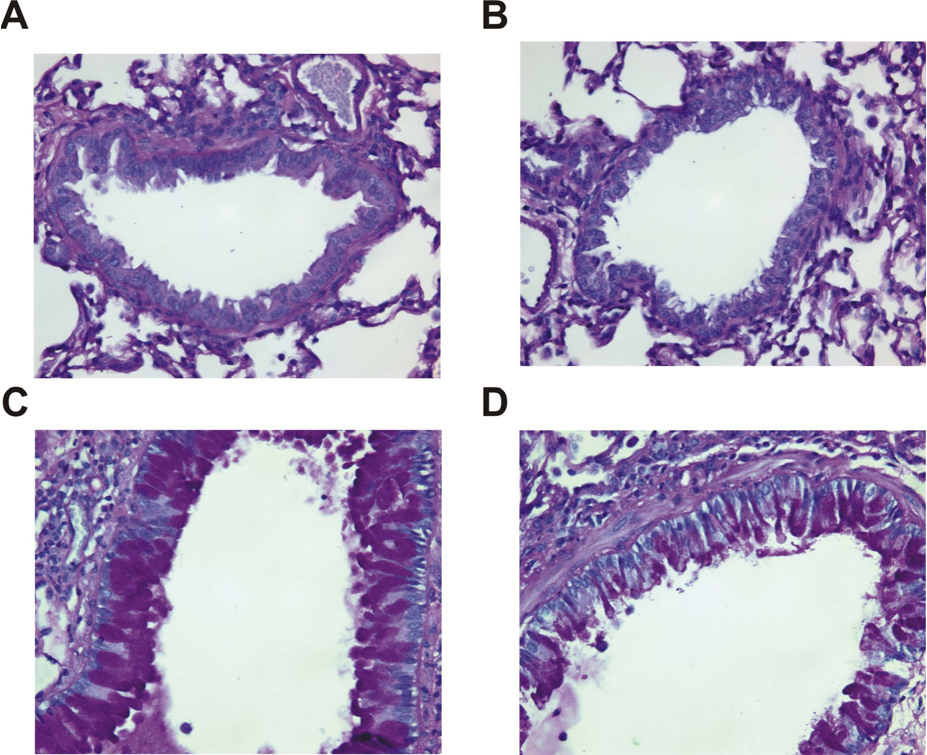
**Histological assessment of mucin staining from PBS or GC frass exposed wild type or PAR-2-deficient Balb/c mice**. Periodic Acid Schiff (PAS) staining of sectioned lungs from PBS (A and B) or GC frass (C and D) treated wild type (A and C) or PAR-2-deficient (B and D) mice. Representative slides are shown of sections from 4-5 mice per group.

To investigate the role of PAR-2 in allergic airway inflammation when administered via the airways, we backcrossed the PAR-2-C57Bl/6 mice into the BALB/c background. Mucosal sensitization of GC frass was performed in wild type and PAR-2-deficient mice by administration of three inhalations of allergen or PBS (Figure 1B). Exposure of wild type mice to GC frass resulted in increased airway responsiveness to cholinergic agents which were significantly decreased in the PAR-2-deficient mice (Figure 3A). Serum IgE levels, which were significantly increased following exposure to GC frass, were also regulated through PAR-2 activation (Figure 3B). Next, we cultured whole lung homogenates from wild type and PAR-2-deficient mice to assess the ability of GC frass to induce Th2 (IL-4, IL-5 and IL-13), Th1 (IFNγ), and Th17 (IL-17A) cytokine production. GC frass significantly increased IL-13 (Figure 3C), IL-5 (Figure 3D), IL-4 (data not shown), and IL-17A (Figure 3E) levels. Importantly, all of these cytokines were significantly reduced in PAR-2-deficient mice compared to wild type mice. In addition, GC frass reduced the levels of IFNγ in both wild type and PAR-2-deficient mice (Figure 3F). Cellular infiltration into the BAL fluid was also significantly different between wild type and PAR-2-deficient mice. Exposure to GC frass resulted in significant recruitment of eosinophils, neutrophils, macrophages and lymphocytes in wild type mice (Table 2). PAR-2-deficient mice had significantly less eosinophils, neutrophils and macrophages in the BAL fluid compared to wild type mice. Histological examination of the wild type or PAR-2-deficient mouse lungs following GC frass treatment showed dense perivascular and peribronchiolar infiltrates compared to PBS treatment (Figure 4). There was a notable decrease in the amount of infiltration of cells into the airways of PAR-2-deficient mice compared to wild type mice. GC frass-treated airways had more mucin than PBS-treated mice (Figure 5). While the number of airways possessing mucin were comparable between wild type and PAR-2-deficient mice (67 ± 2 airways in wild type mice and 69 ± 1), the amount of cells expressing mucin was different. In the airways of wild type mice, 90.3 ± 3.2% of the cells expressed mucin while in the PAR-2-deficient mice only 72.5 ± 5% of the cells expressed mucin (p = 0.013 as determined by ANOVA). Collectively, these data indicate a role for PAR-2 in mediating GC frass-induced allergic airway inflammation.

**Table 2:** Differential cell count in BAL fluid of wild type and PAR-2-deficient mice.

	Mac	Epi	Eos	Neut	Lymph
**Balb/c-PBS**	0.9 ± 0.4	1.8 ± 0.6	0	0.04 ± 0.02	0.07 ± 0.06

**PAR2-/- PBS**	2.0 ± 0.7	3.0 ± 1.2	0	0.02 ± 0.02	0.04 ± 0.02

**Balb/c-frass**	7.8 ± 0.9*	5.2 ± 1.1	4.7 ± 1.3*	5.9 ± 3.9*	2.9 ± 0.7*

**PAR2-/- frass**	3.8 ± 0.9**	3.1 ± 1.2	1.7 ± 0.8**	0.8 ± 0.2**	2.3 ± 0.9*

Wild type (Balb/c) or PAR-2-deficient mice were given intratracheal inhalations of PBS (40 μl) or GC frass (40 μg/40 μl) on days 0, 7, and 14. On day 17, BAL fluid was harvested and differential cell counts performed. These data represent 4 mice per group and are expressed as mean ± SEM of cell number × 10^4^. Cell counts were statistical significance between GC frass and PBS exposed mice (*p < 0.05, as determined by ANOVA). In addition, statistical differences were found between Balb/c and PAR-2-deficient mice exposed to GC frass (**p < 0.05).

### GC frass proteases are crucial in the initiation phase of experimental asthma

**Figure 6:**
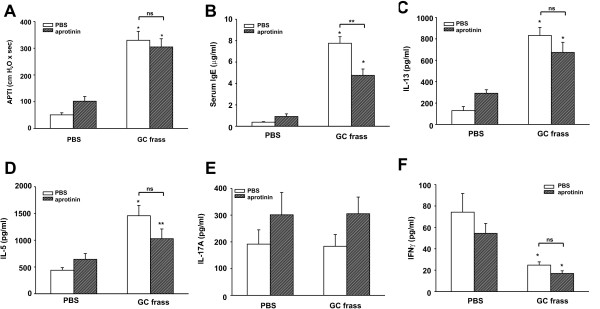
**GC frass proteases do not regulate airway inflammation and airway hyperresponsiveness**. BALB/c mice were sensitized on day 0 and 7 with an intraperitoneal injection of PBS, aprotinin-treated PBS, GC frass, or GC frass pretreated with aprotinin (10 μg/ml) and then bound to alum. On days 14 and 19, an intratracheal inhalation was performed using PBS, PBS pretreated with aprotinin, GC frass (40 μg) or GC frass pretreated with aprotinin. On day 22, mice were anesthetized acetylcholine was injected after establishment of a stable airway pressure. In all cases the data are expressed as mean ± SEM and represent 8 mice per group and statistical significance determined by ANOVA. A. AHR was measured as airway pressure time index (APTI) in cm-H_2_O × sec ^-1 ^(compared to PBS * p < 0.001). B. Serum IgE levels were analyzed by ELISA (compared to PBS *p < 0.001, compared to GC frass **p < 0.002). C-F. Lungs from the mice were excised, and cells dissociated and maintained in a single suspension culture for 3 days in the presence of Con A (10 μg/ml). C. IL-5 levels (compared to PBS *p < 0.05). D. IL-13 levels (compared to PBS *p < 0.001). E. IL-17A levels. F. IFNγ levels (compared to PBS *p < 0.05).

**Figure 7:**
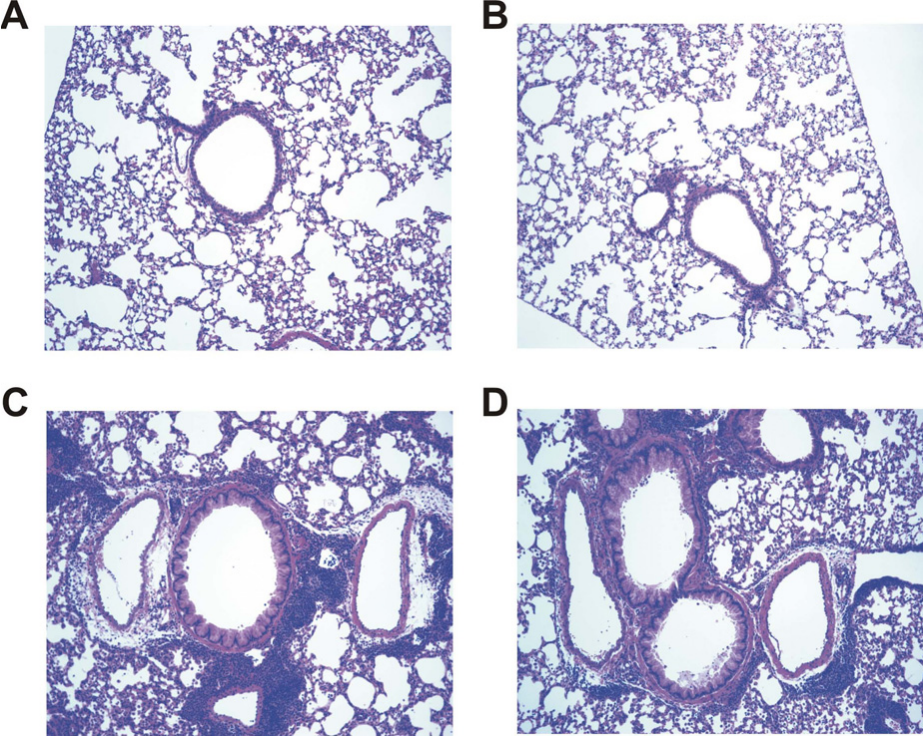
**Histological assessment of lung sections from mice exposed to GC frass or protease-depleted GC frass**. Haematoxylin and eosin (H & E) staining of sectioned lungs mice exposed to PBS (A), aprotinin-treated PBS (B), GC frass (C) or protease-depleted GC frass (D). Representative slides are shown of sections from 8 mice per group.

**Figure 8:**
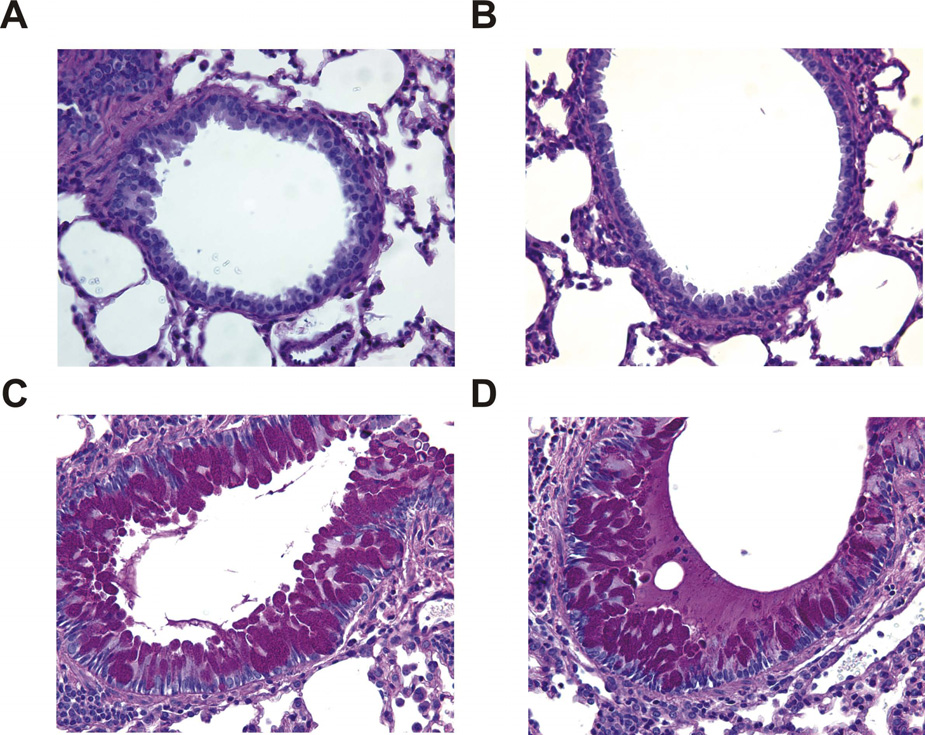
**Histological assessment of mucin staining from mice exposed to GC frass or protease-depleted GC frass**. Periodic Acid Schiff (PAS) staining of sectioned lungs from mice exposed to PBS (A), aprotinin-treated PBS (B), GC frass (C) or protease-depleted GC frass (D). Representative slides are shown of sections from 8 mice per group.

Our recently published data showed that mucosal sensitization to protease-depleted GC frass resulted in decreased airway hyperresponsiveness and decreased mucin production [8]. To define the role of GC frass proteases in modulating allergic airway disease, we asked if protease-depleted GC frass would play a role in systemic allergic sensitization. To do this, mice were sensitized with GC frass or protease-depleted GC frass bound to alum via intraperitoneal injection (Figure 1A). These mice were then challenged with an intratracheal inhalation of either GC frass or protease-depleted GC frass on day 14 and 19. On day 22, mice were sacrificed and allergic airway inflammation and airway hyperresponsiveness were assessed. As expected, GC frass induced airway hyperreactivity to acetylcholine, serum IgE levels, Th2 cytokines, cellular influx and mucin production (Figure 6). Importantly, removal of GC frass proteases had no effect on airway hyperreactivity (Figure 6A), Th2 cytokine production (Figures 6C and 6D), or Th1 cytokine production (Figure 6F). IL-17 was not induced in these mice in this sensitization protocol by GC frass (Figure 6E). We also found that removal of proteases significantly decreased GC frass-induced serum IgE levels (Figure 6B). GC frass induced significant recruitment of macrophages, eosinophils, neutrophils and lymphocytes into the BAL fluid; however these levels were unaltered when mice were sensitized to protease-depleted GC frass (data not shown). Histological examination of the mouse lungs following GC frass treatment showed dense perivascular and peribronchiolar infiltrates compared to PBS treatment (Figure 7). Depletion of proteases from GC frass had little effect on the amount of perivascular and peribronchiolar infiltrates compared to GC frass treatment. While GC frass significantly increased the amount of mucin, we could not detect any significant differences in the amount of mucin between GC frass and protease-depleted GC frass (Figure 8). Collectively, these data show that the proteases associated with allergens do not regulate allergic sensitization when they are administered systemically, highlighting the importance of the resident airway cells (airway epithelium and alveolar macrophages) in the initiation of allergic airway disease.

## Discussion

In this study, we investigated the role of PAR-2 in mediating allergic airway inflammation using a real world allergen. Our initial experiments showed that GC frass-induced allergic airway inflammation was unaltered in PAR-2-deficient mice compared to wild type mice. At first blush, this might suggest that GC frass proteases were altering allergic airway inflammation independent of PAR-2. However, a crucial difference between the studies in BALB/c mice and C57Bl/6 mice was the method of sensitization (mucosal vs. systemic) and the use of the Th2 stimulator alum. The commercially available PAR-2-knockout mice were in the C57Bl/6 background; one that we have previously shown is not highly responsive to mucosal sensitization [8,27], and thus required systemic sensitization with alum. To directly study the effect of GC frass protease on mucosal sensitization of PAR-2-deficient mice, we backcrossed the PAR-2-deficient mice onto the BALB/c strain. Mucosal exposure of GC frass to PAR-2-deficient BALB/c mice resulted in diminution of all aspects of allergic airway inflammation compared to wild type mice. Together these data confirmed the importance of PAR-2 in the initiation of allergic airway disease when sensitization occurred at the mucosal surface.

In an attempt to clarify the importance of proteases administered at the mucosa compared to systemically, we compared GC frass with and without protease activity during systemic sensitization. We found that systemic sensitization with protease-depleted GC frass did not alter airway inflammation or AHR compared to protease-containing GC frass, suggesting that removal of proteases are not important when sensitization occurs systemically. However, in order to perform these experiments we had to bind the allergen to alum, so we cannot fully rule out the possibility that alum may overwhelm the immunostimulatory effects of protease activity in GC frass. Nonetheless, the idea that proteases would play a contributory role at the epithelium fits in well with literature demonstrating a role for PAR-2 and proteases in epithelial cell activation. Cockroach [30,32], HDM[3] and mold [20] proteases have all been shown to induce IL-8 expression from cultured airway epithelial cells in a PAR-2-dependent manner. The idea that alteration of the epithelium can initiate allergic airway disease has been eloquently shown in the studies by Pantano et. al. [33]. They showed that upregulation of NF-κB in the airway epithelium of transgenic mice without any other stimulus was sufficient for AHR and smooth muscle thickening. These data suggest the crucial role of the airway epithelium in asthma pathogenesis, and lends support to our data that proteases administered via the airways can ultimately modulate airway inflammation and AHR.

Interestingly protease-depleted GC frass bound to alum and administered by intraperitoneal injection decreased total serum IgE levels. We did not assay for GC frass-specific IgE or aprotinin-specific IgE in this study. The regulation of serum IgE levels may be due to protease activity or may be a non-specific effect. Aprotinin was widely used to prevent bleeding and reduce blood transfusions after surgical procedures until it was shown that the use of aprotinin was associated with doubling the risk of renal failure in patients undergoing heart surgery [34]. There is at least one report showing a severe anaphylactic reaction with highly elevated IgE levels after a single exposure to aprotinin in a young child [35]. In our studies, aprotinin alone increased IgE; however aprotinin-treated GC frass was shown to decrease serum IgE. Since aprotinin-treated GC frass administered systemically had no effects other than on serum IgE levels, we have not followed up on this finding.

C57Bl/6 mice have been shown to be the least responsive to systemic OVA allergen exposure [36,37] and were not very responsive to GC frass when exposed at the mucosa [38]. However it was recently demonstrated that HDM allergen induced Th2 cytokines, eosinophil infiltration and AHR in C57Bl/6 mice [39]. It is likely that OVA, HDM and GC frass differ in their adjuvant properties or even in their pathogen-associated molecular patterns (PAMPs), both of which can alter allergenicity. In addition, there is new information suggesting that the colonization of the gastrointestinal tract of mice can play a role in the immune response [40]. These exciting data could potentially explain slightly different immunological responses found in different labs. At this point it is unclear why different strains of mice are more or less susceptible to allergic airway inflammation. It has been shown that certain mouse strains have a genetic predisposition to a Th1 or Th2 response which appears to be independent of provocation. For example, C57Bl/6 mice tend to mount Th1 immune responses, while BALB/c mice tend to mount a Th2 response [41] as evidenced by the finding that C57Bl/6 mice can clear Leishmania major infection in a Th1-mediated fashion, while BALB/c mice have a Th2 response [42]. The predisposition to mount either a Th1 or Th2 response may also be a factor in allergic asthma in humans as well.

We cannot rule out the possibility that asthma genes surround the PAR-2 locus that may have been introduced into the BALB/c mice when we backcrossed the PAR-2 locus into that strain of mice. All genes encoding PARs map to chromosome 13D2 in mouse and 5q13 in humans [43]. A very recent report showed that chromosome 13 in mice played a role in AHR and they found 29 candidate genes of interest [44]. Human chromosome 5 has also been implicated to contribute to asthma as both IgE and IL-4 are found on 5q31.1 [45]. However, since protease-depleted GC frass decreased AHR and mucin production in wild type mice [8] similar to our findings with the PAR-2 backcrossed mice, we feel that it is unlikely that our effects are due solely to asthma genes surrounding the PAR-2 locus.

This is the first to address the role of PAR-2 in mediating allergic airway inflammation and AHR using a physiologically relevant allergen administered in a physiologically relevant manner. A few groups have explored the role of PAR-2 in mediating airway inflammation and AHR using either PAR-2-deficient mice or PAR-2 activating peptides. Schmidlin et. al. showed that systemic sensitization to OVA in PAR-2-deficient mice resulted in decreased cellular infiltration and a trend towards decreased airway hyperresponsiveness [23]. Another group sensitized mice with OVA and then challenged with OVA in the presence or absence of PAR-2 activating peptides showed an increase in airway inflammation and reactivity [46]. In contrast, it was also shown that activation of PAR-2 reduced airway inflammation in a rabbit model of experimental asthma [47]. In that study, they sensitized mice to pollen and then aerosol challenged rabbits with allergen in the presence or absence of PAR-2 activating peptides and found that PAR-2 activation decreased airway reactivity. The conflicting effects of PAR-2 could be due to timing of the activation of PAR-2, for example in both studies PAR-2 was administered during the effector phase of allergic disease. Using a physiologically relevant method of exposure, we highlight the importance of mucosal sensitization in this process. These data also suggest the role of allergen-derived proteases as an adjuvant in the development of allergic airway disease.

## List of Abbreviations

APTI: airway pressure time index; BAL: bronchoalveolar lavage; GC: German cockroach; HDM: house dust mite; IL: interleukin; OVA: ovalbumin; PAR: protease-activating receptor; PAMP: pathogen-associated molecular pattern.

## Competing interests

The authors declare that they have no competing interests.

## Authors' contributions

KP designed and performed the experiments and drafted the manuscript. JRL performed the animal work and the microscopy. PZ performed the immunoassays. MWK participated in the design of the study and helped draft the manuscript. All authors read and approved the final manuscript.
